# Pneumatic-type surgical robot end-effector for laparoscopic surgical-operation-by-wire

**DOI:** 10.1186/1475-925X-13-130

**Published:** 2014-09-05

**Authors:** Chiwon Lee, Woo Jung Park, Myungjoon Kim, Seungwoo Noh, Chiyul Yoon, Choonghee Lee, Youdan Kim, Hyeon Hoe Kim, Hee Chan Kim, Sungwan Kim

**Affiliations:** Interdisciplinary Program for Bioengineering, Graduate School, Seoul National University, Seoul, 110-744 South Korea; Department of Mechanical & Aerospace Engineering, Seoul National University College of Engineering, Seoul, 151-742 South Korea; Department of Urology, Seoul National University Hospital, Seoul, 110-744 South Korea; Department of Biomedical Engineering, Seoul National University College of Medicine, Seoul, 110-799 South Korea; Institute of Medical and Biological Engineering, Seoul National University, Seoul, 151-742 South Korea

**Keywords:** Laparoscopic surgical robot system, Minimally invasive surgery (MIS), End-effector of surgical robot, Surgical-operation-by-wire (SOBW), Pneumatic gripping system, Hands-on-throttle-and-stick (HOTAS)

## Abstract

**Background:**

Although minimally invasive surgery (MIS) affords several advantages compared to conventional open surgery, robotic MIS systems still have many limitations. One of the limitations is the non-uniform gripping force due to mechanical strings of the existing systems. To overcome this limitation, a surgical instrument with a pneumatic gripping system consisting of a compressor, catheter balloon, micro motor, and other parts is developed.

**Method:**

This study aims to implement a surgical instrument with a pneumatic gripping system and pitching/yawing joints using micro motors and without mechanical strings based on the surgical-operation-by-wire (SOBW) concept. A 6-axis external arm for increasing degrees of freedom (DOFs) is integrated with the surgical instrument using LabVIEW® for laparoscopic procedures. The gripping force is measured over a wide range of pressures and compared with the simulated ideal step function. Furthermore, a kinematic analysis is conducted. To validate and evaluate the system’s clinical applicability, a simple peg task experiment and workspace identification experiment are performed with five novice volunteers using the fundamentals of laparoscopic surgery (FLS) board kit. The master interface of the proposed system employs the hands-on-throttle-and-stick (HOTAS) controller used in aerospace engineering. To develop an improved HOTAS (iHOTAS) controller, 6-axis force/torque sensor was integrated in the special housing.

**Results:**

The mean gripping force (after 1,000 repetitions) at a pressure of 0.3 MPa was measured to be 5.8 N. The reaction time was found to be 0.4 s, which is almost real-time. All novice volunteers could complete the simple peg task within a mean time of 176 s, and none of them exceeded the 300 s cut-off time. The system’s workspace was calculated to be 11,157.0 cm^3^.

**Conclusions:**

The proposed pneumatic gripping system provides a force consistent with that of other robotic MIS systems. It provides near real-time control. It is more durable than the existing other surgical robot systems. Its workspace is sufficient for clinical surgery. Therefore, the proposed system is expected to be widely used for laparoscopic robotic surgery. This research using iHOTAS will be applied to the tactile force feedback system for surgeon’s safe operation.

## Introduction

Minimally invasive surgery (MIS) using conventional laparoscopic tools has emerged as a new paradigm for surgical operation because it offers many advantages such as smaller incision, reduced hemorrhaging, less pain, reduced exposure of internal organs to possible external contaminants, faster recovery, and short-term hospitalization period compared to conventional open surgery. MIS is thus greatly beneficial to patients. However, it suffers from some disadvantages: only skilled surgeons can perform non-robotic surgery, surgeons are not provided with haptic feedback, surgeries take longer compared with open surgery, suturing is difficult, and the degree of freedom (DOF) of the end-effector is less sufficient to perform surgery [1–3]. Robotic laparoscopic surgery has thus been rapidly developed as a means to resolve the issues faced with open surgery and non-robotic surgery [3–6].

Over the last decade, more than 1.5 million laparoscopic surgical operations, including gynecologic, cardiac, urology, thoracic, head & neck, and general surgery, have been performed worldwide using the da Vinci robot (Intuitive Surgical, Inc., Sunnyvale, CA, USA), a market-leading surgical robot system [7]. Many research groups have aimed to improve the da Vinci system or to propose novel surgical robot systems. A surgical robot end-effector with a new joint mechanism for large force, accurate motion, and preventing joint hysteresis has been proposed [8]. Raven-II, a platform for collaborative research on advances in laparoscopic surgery, has been reported; this system has a 2-DOF spherical positioning mechanism and a 4-DOF instrument using mechanical strings [5]. A surgical intervention end-effector with integrated stereo vision has been developed [9]; this system’s end-effector is inserted through a single 15-mm access port, and the end-effector’s actuation unit is bulky. A single port laparoscopic robot where grippers and elbow/shoulder are decoupled has been developed [10]. This system is well integrated with decoupled joints and actuated for complex movement. However, one drawback of this system is the bigger diameter (18-mm) which needs to be reduced for small incision, too. These research groups have aimed to imitate users’ wrist motions, such as pitching, yawing, rolling, and gripping motions, within an approximately 8-mm diameter as same diameter of da Vinci’s EndoWrist. However, their proposed devices suffer from several drawbacks, including long peg task time, coupling with several moving joints, and bulky size [5, 8–10]. A gear driven mechanism is a general method being applied to conventional robot system, but it is very hard to be directly applicable to surgical robot end-effector system which has 8 ~ 10-mm of diameter. Some efforts using a gear system are found in [10], but the diameter is bigger than the above range. So, da Vinci system is a representative surgical robot system, but it is using mechanical string & pulley to keep the diameter within 8-mm and to sterilize. In addition, securing sufficient gripping force is an important issue in a laparoscopic surgical robot system. However, the da Vinci surgical robot system’s EndoWrist is reported to have different gripping forces for different wrist postures [11]. These limitations are considered to arise from the joints of the gripping motion, which is used for generating driving force, being coupled with other joints through mechanical strings. These problems similarly arise in aerospace engineering, where a pilot’s control stick is connected to the wing’s control surfaces through mechanical strings, cables, or many mechanical parts [12–14]. In this field, most of these problems are resolved by adopting a fly-by-wire (FBW) system that directly drives the wing control parts, such as the control surfaces, using the ends of the wing’s actuators and eliminates the need for mechanical strings [15, 16]. In the airplane with the FBW system, almost all mechanical connection parts for wing control are replaced with electrical wire for reliable control [17]. This aerospace technology has inspired a novel concept; surgical-operation-by-wire (SOBW).

In the medical field, the present study aims to develop a SOBW concept. The SOBW, which is first defined in this study, is a concept which replaces mechanical strings with electrical wire in the surgical robot system. Similar concept of SOBW is revealed in the existing surgical robot system; da Vinci robot which could be regarded as a semi-SOBW system because it uses many mechanical strings in internal parts. In the proposed surgical robot system, all mechanical strings are therefore removed and all joints are driven directly by actuators such as alternative current (AC) servo motors in the external arm and micro motors in surgical instrument with a diameter of 8-mm for full SOBW system. However, previous studies have shown that motions such as pitching, yawing, rolling, and gripping cannot be integrated into an 8-mm diameter [10, 18–20]. Furthermore, while a micro motor is appropriate for moving the joint, it cannot provide sufficient gripping force. So, it is necessary to develop a new gripping system. A new type of pneumatic end-effector is developed for the gripping motion. The gripping force is adjustable by the controlling pressure using a pneumatic system consisting of a compressor, air pump, 3-way solenoid valves (SVs), speed controller, pressure controller, and catheter balloon which tolerates high pressure for clinical use [21]. This gripping system is decoupled from the external arm and the pitching/yawing joint, unlike existing laparoscopic surgical robots. Therefore, sufficient gripping force is obtained and maintained regardless of the end-effector’s different postures. Through repeated gripping experiments, the surgical instrument’s durability is verified. In this study, the surgical robot system adopts a hands-on-throttle-and-stick (HOTAS) controller for the surgeon’s control interface. HOTAS is used for flight control in the aerospace field, and it can control hundreds of functions and provide feedback to the pilot about flight conditions. Similarly, it can be used to help surgeons perform many surgical operations, and it can be easily applied to force feedback research. The 6-axis robot is integrated with the proposed surgical instrument for a surgical peg task with the aim of examining the clinical applicability of the proposed system. This novel surgical robot system can be widely used for laparoscopic robotic surgery.

## System description

**Figure 1:**
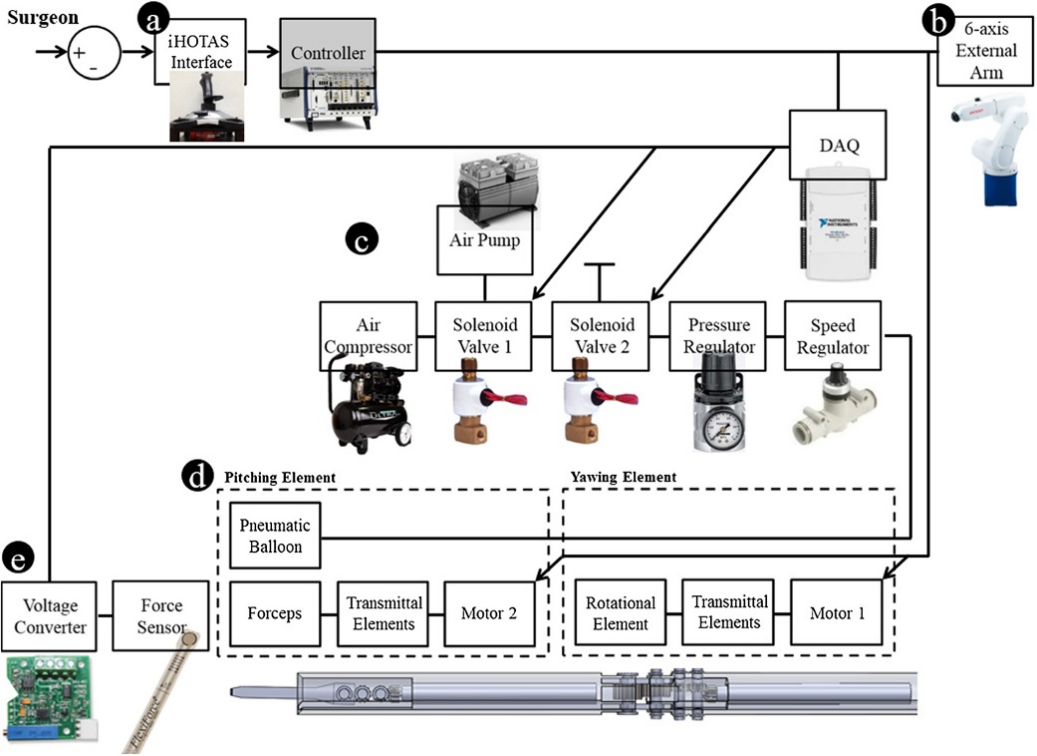
**Control block diagram and experimental flow of the overall system. (a)** Interface for surgeon. **(b)** External arm. **(c)** Pneumatic gripper system. **(d)** Surgical instrument. **(e)** Gripping force measurement system using data acquisition (DAQ) board. All hardware is controlled using the LabVIEW® software based on the state machine structure.

**Figure 2:**
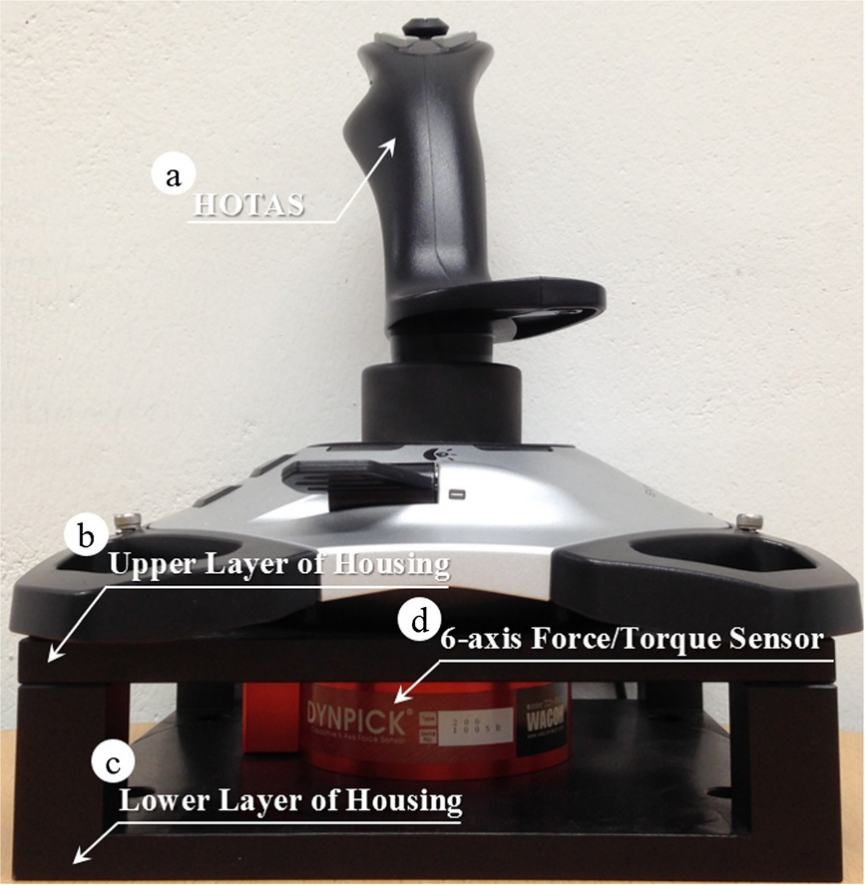
**Improved hands-on-throttle-and-stick (iHOTAS). (a)** Conventional HOTAS controller. **(b)** Upper layer of the special housing. **(c)** Lower layer of the special housing. **(d)** 6-axis force/torque sensor. All the screws in the special housing assembly were tightly secured to ensure the precise measurement. The improved HOTAS (iHOTAS) controller was used to perform translational movement.

A control flow of the entire system is depicted in Figure 1. The system consists of the HOTAS interface that can reflect the surgeon’s decision, the control 6-axis external robot arm, and the surgical instrument with the pneumatic control system. To improve the function of the HOTAS controller, a 6-axis force/torque sensor (Dynpick, Wacoh-Tech Inc., Takaoka City, Futatsuka, Japan) was attached to the bottom in a special housing as shown in Figure 2. A threaded upper and lower assembly parts of 6-axis force/torque sensor were attached with special housing’s upper and lower layer, respectively. All the screws in the special housing assembly were tightly secured to ensure the precise measurement. The improved HOTAS (iHOTAS) controller was used to perform translational movement. Hardware related to the surgical robot system were integrated with LabVIEW® and PXIe controller (LabVIEW® 2013, PXIe-8135 & 1062Q, National Instruments, Austin, TX, USA, Used valid license). Air flow control of the pneumatic system using two SVs was executed by a data acquisition board (USB-6212 DAQ, National Instruments, Austin, TX, USA). The pitching/yawing joints of the surgical instrument were controlled by a micro motor and a motor controller (EC-4 motor, EPOS2 controller, Maxon Motor, Brünigstrasse, Sachseln, Switzerland).

### Overview

The proposed surgical robot system could be divided into two parts: external arm and surgical instrument. The former could perform 6-DOF movements including translational motion, fulcrum point motion, and the surgical instrument’s rolling motion. The latter could perform 2-DOF movements such as the yawing and pitching motions and gripping motion. A pneumatic gripper was installed at the end of the surgical instrument. Because the external arm and the surgical instrument were decoupled, unlike in almost all other surgical robot systems [5, 8, 22], the surgical instrument could be detached from the external arm and be easily replaced during surgery. The executing force of the surgical robot system was generated by 6 AC servo motors (VS-6556G, DENSO, Kariya, Aichi Prefecture, Japan), 2 micro motors (EC-4 & 280:1 ∅4 planetary gearhead, Maxon Motor, Brünigstrasse, Sachseln, Switzerland), and a pneumatic compressor.

### External arm

**Figure 3:**
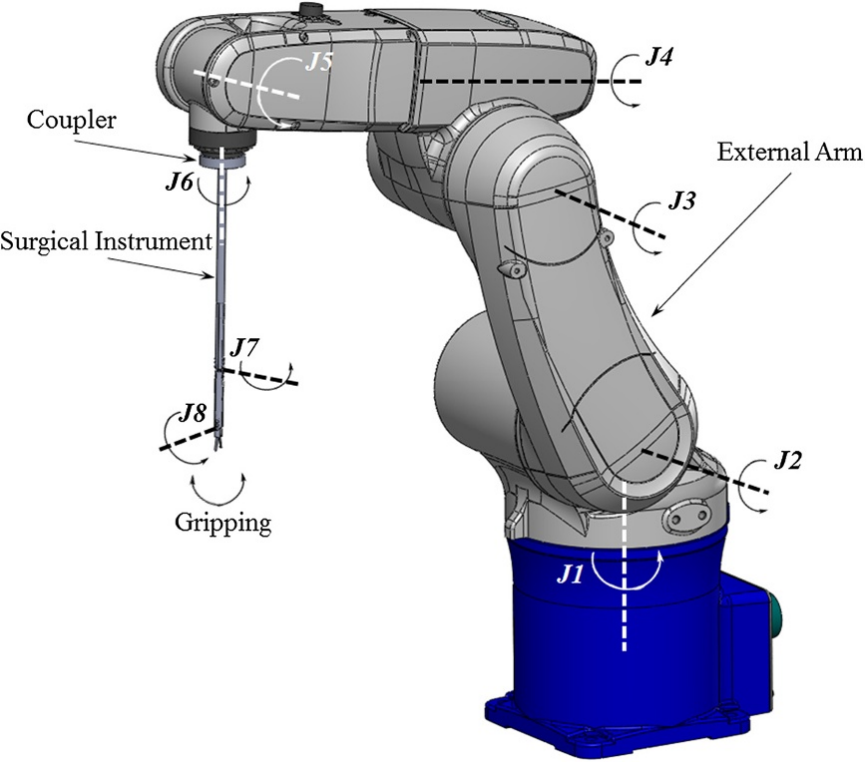
**Conceptual design of the surgical robot system.**

For translational motion, fulcrum point motion, and the surgical instrument’s rolling motion, a 6-axis external arm (VS-6556G, DENSO, Kariya, Aichi Prefecture, Japan) was utilized. In Figure 3, *J1-J5* are complexly involved with the translational motion and fulcrum point motion. *J6* independently executes the surgical instrument’s rolling motion. The complex movements of *J1-J5* were controlled by tool coordinates. The tool coordinates set the external arm’s origin to the origin of the end-effector. The external arm moves on the basis of the fulcrum point and translational motion according to the user’s iHOTAS control.

### Surgical instrument

**Figure 4:**
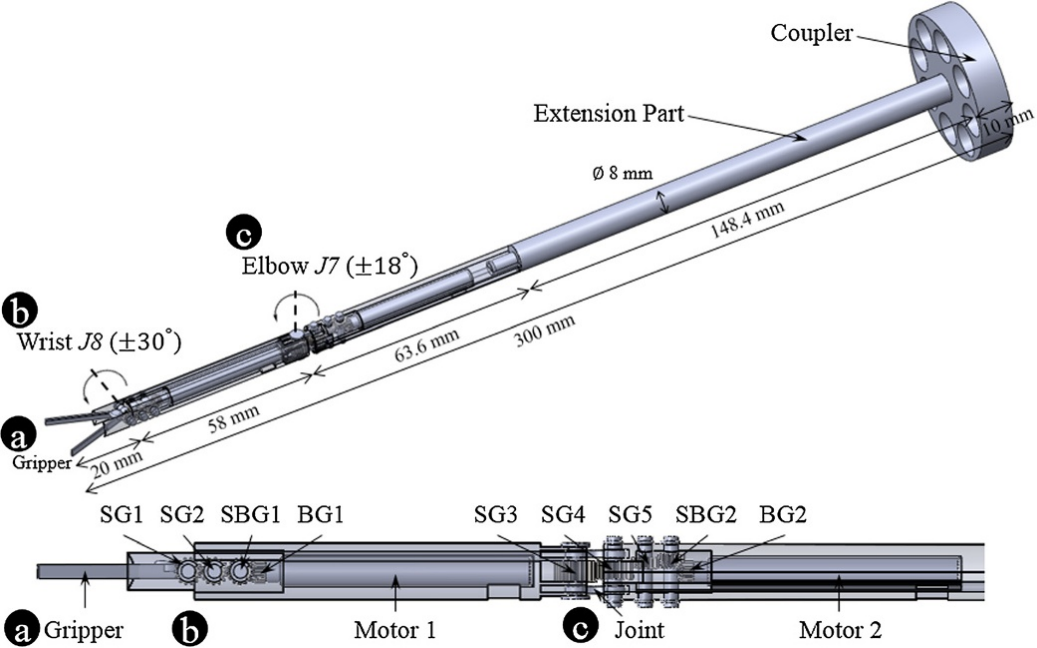
**Design of surgical instrument. (a)** Pneumatic gripper. **(b)** Wrist joint. **(c)** Elbow joint. Several gears, outer shells, micro motors, and joint link are assembled. This instrument performs elbow, wrist, and gripping motions. The surgical instrument’s length and outer diameter is 300-mm and 8-mm, respectively. Abbreviation: spur gear (SG), spur and bevel gear (SBG), and bevel gear (BG).

**Figure 5:**
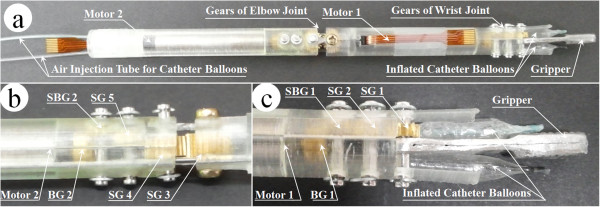
**Actual surgical instrument. (a)** Entire surgical instrument. **(b)** Zoom in for elbow joint. **(c)** Zoom in for wrist joint and closed gripper by inflated catheter balloons. The position of the micro motors, several gears, and gripper are presented in this figure. The inflated catheter balloons make gripper close the gripper’s tips by Newton’s 3rd law.

**Figure 6:**
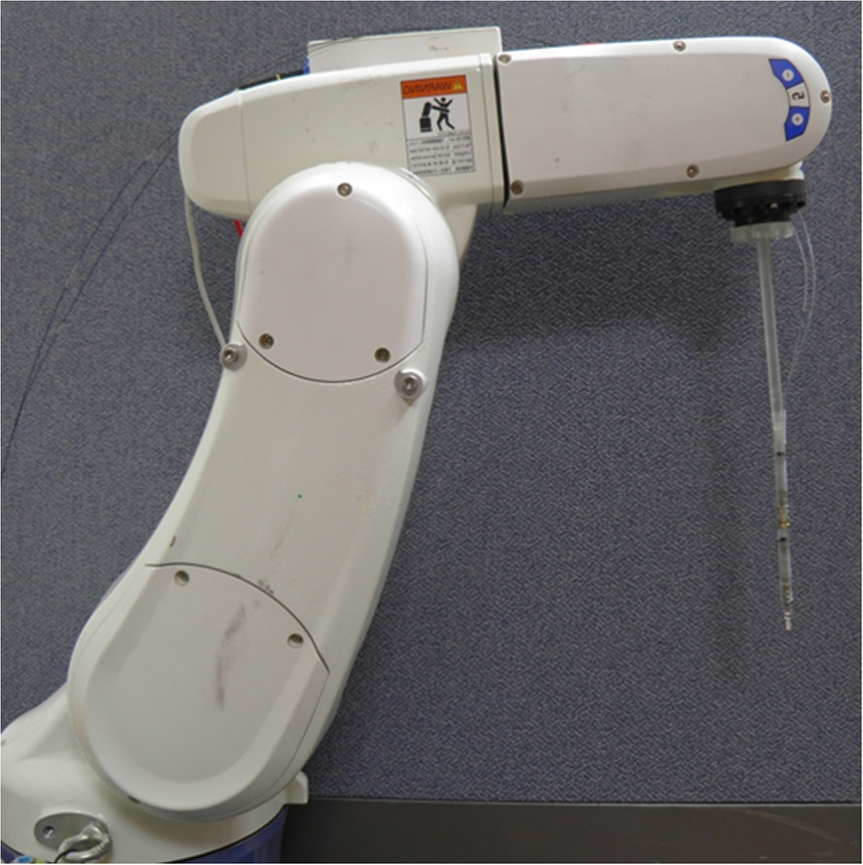
**Assembled surgical instrument and external arm.**

The flexion/extension motions of the wrist were performed using the surgical instrument’s pitching motion in *J8*. The radial/ulnar deviation motions of the wrist could be overcome by a combination of the surgical instrument’s pitching motion (*J8*) and rolling motion (*J6*, external arm). The flexion and supination motions of the elbow could be compensated by the surgical instrument’s yawing motion (*J7*) and rolling motion (*J6*, external arm), as shown in Figure 3. The ranges of elbow and wrist joint were 36° and 60°, respectively. An elbow joint would be helpful in decreasing the probability of the surgical instruments’ collision with the outside of the abdominal cavity [8]. The driving force of the surgical instrument’s pitching and yawing motion was not generated using mechanical strings, as in other systems [8, 22, 23]. Micro motors were used to perform pitching and yawing motions in the outer shells, as shown in Figures 4 and 5. The surgical instrument which removed coupler and extension part from Figures 4 and 6 was shown in Figure 5. This figure represented the actual gripper, elbow joint, and wrist joint in detail. The gripper could be closed by inflating catheter balloons as shown in Figure 5-(c). Outer shells were manufactured using a 3D printer (Form 1, Formlabs, Somerville, MA, USA) to the nearest sub-millimeter resolution and to assemble several parts such as micro motors, gears, and joint links. The surgical instrument was 300-mm long for surgical usability. The outer diameter was 8-mm, the same as that of the da Vinci surgical robot system’s EndoWrist, for MIS. In addition, the driving force of gripping motion was generated from the pneumatic system’s compressor. The pneumatic gripping system enabled complex yawing and pitching movements, provided sufficient gripping force, and was decoupled from the external arm within an 8-mm outer diameter. The pitching motion could be directly actuated by the micro motor if the micro motor was able to tolerate weight of the gripper and the yawing motion could be achieved when the micro motor could tolerate the weights of elbow part, which was consist of the gripper, one micro motor, five gears, and outer shell. The weight of the whole surgical instrument was 36 g. The weights of the driving parts (elbow and wrist part) of surgical instrument and extension part with coupler were 15 g and 21 g, respectively. As for the elbow part in driving parts, it only occupied 7 g. Since the micro motor had the torque of 0.0473 N · m (0.4827 kgf · cm = 482.7gf · cm, the efficiency of the micro motor and planetary gearhead were considered) by using 280:1 of gear rate, it was sufficiently able to tolerate the weights as mentioned above.

### Pneumatic gripper system

**Figure 7:**
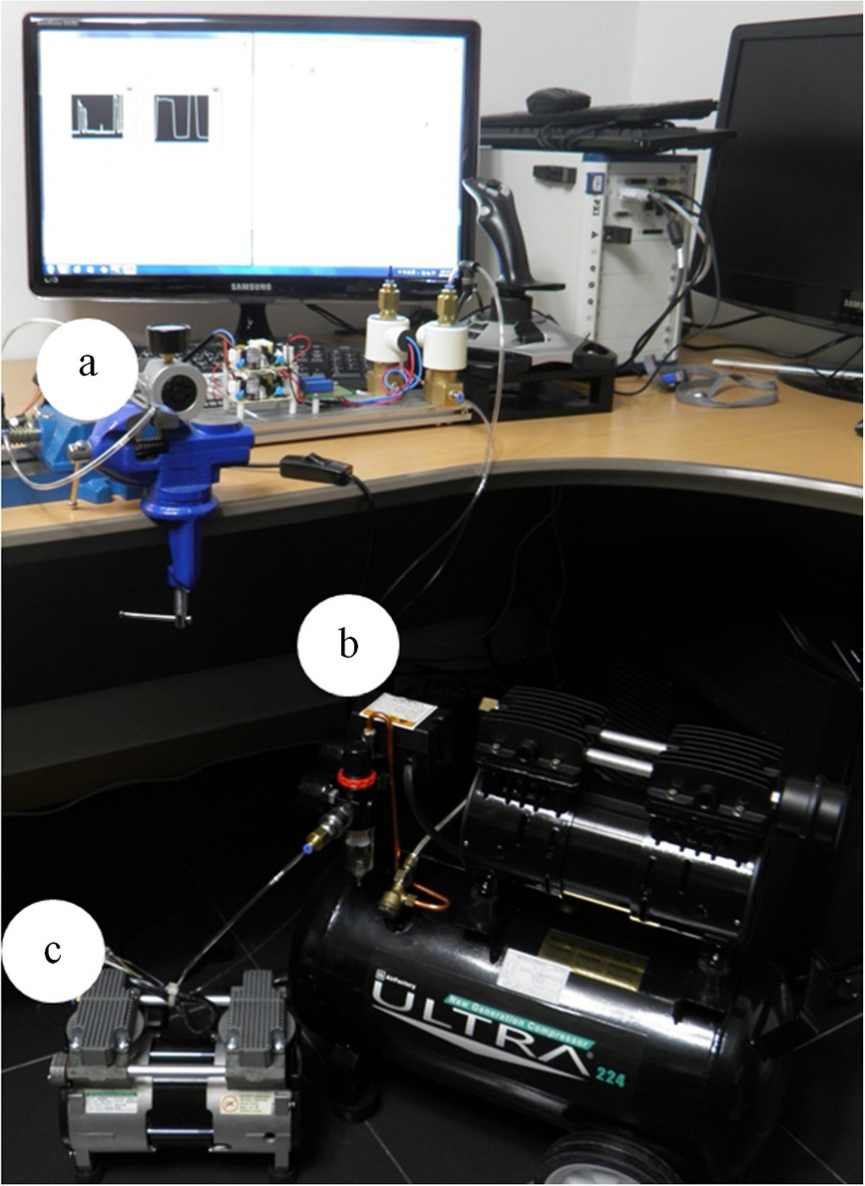
**Pneumatic hardware system. (a)** Solenoid valves, speed regulator, and pressure regulator control the compressed air. **(b)** Air compressor pumps compressed air into the catheter balloon. **(c)** Air pump sucks compressed air out of the catheter balloon.

**Figure 8:**
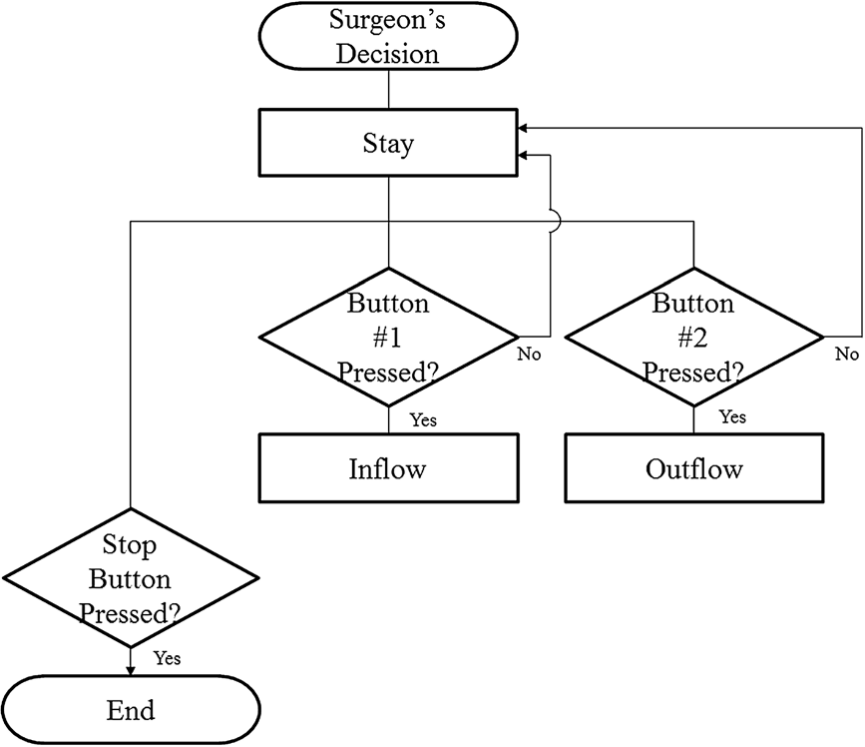
**Diagram of valve control algorithm.** Three valve statuses can be controlled by the surgeon.

**Figure 9:**
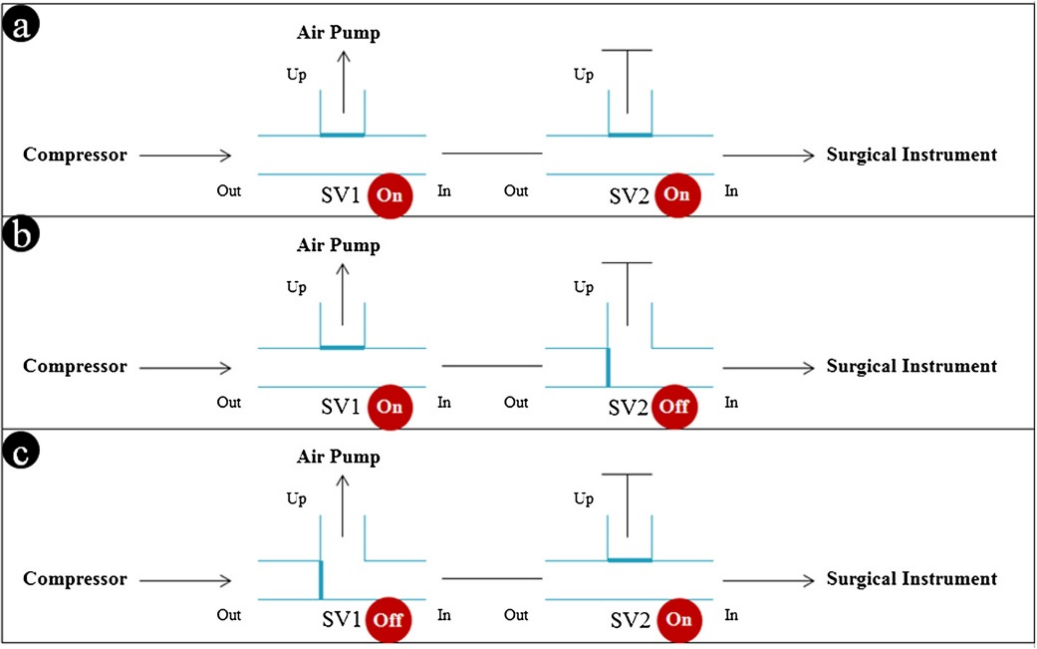
**Compressed air flow by valve mechanism. (a)** Inflow. **(b)** Stay. **(c)** Outflow. Three compressed air flow statuses are controlled by SV1 and SV2 between the compressor and the catheter balloons. Abbreviation: solenoid valve (SV).

The gripping motion was achieved by inflating and deflating the catheter balloon. The air compressor (ULTRA 224, AirFactory, Seoul, South Korea) and air pump were used to pump compressed air into and suck the same out of the catheter balloon, respectively. The compressed air was controlled using SVs, a speed regulator, and a pressure regulator, as shown in Figure 7. The surgeon’s decision was reflected by the pneumatic gripper system, as shown in Figure 8. To control the gripping motion, two SVs were controlled with one of three statuses: inflow, stay, and outflow. In Figure 9, compressed air flowed from the compressor to the surgical instrument’s catheter balloon via SV1 and SV2 for the inflow status (SV1 and SV2: On). It could inflate the catheter balloon to close the gripper. Compressed air could not be flowed into the surgical instrument and halted at SV2 for the stay status (SV1: On, SV2: Off). For opening the gripper in the outflow status, SV1 and SV2 were turned off and on, respectively. At this time, the remaining compressed air in the surgical instrument flowed to the atmosphere by the air pump.

### Forward kinematics of the system

**Figure 10:**
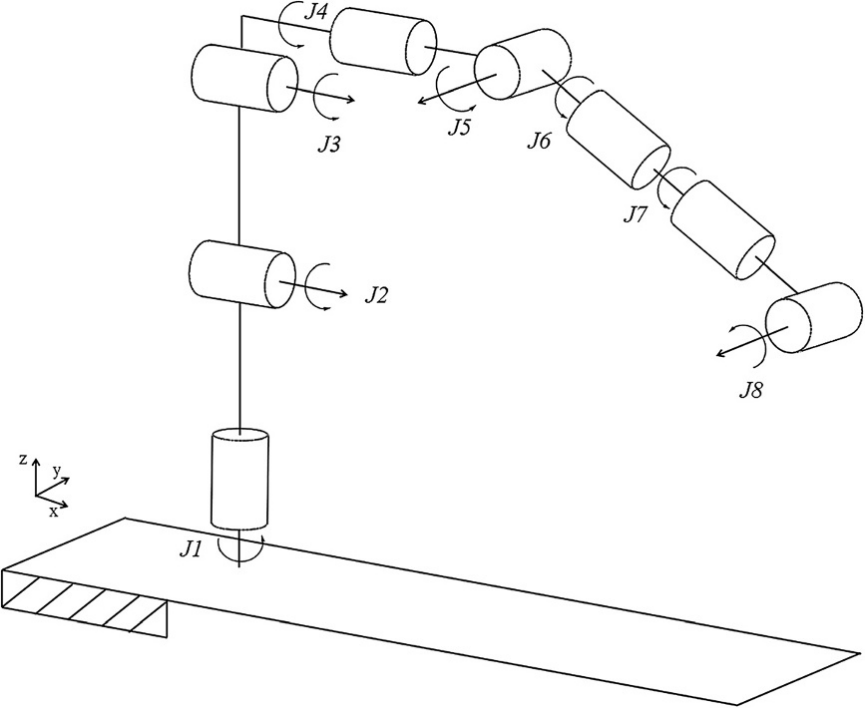
**Kinematic structure of the system.**

Figure 10 shows the kinematic structure of the entire system, except for the gripping motion. *J1-J6* and *J7-J8* represent the external arm parts and surgical instrument, respectively. Table 1 shows the Denavit-Hartenberg (D-H) parameters of this system. With reference to Table 1, each joint’s information such as operational angle and other information could be confirmed. These homogeneous transformation matrices are inferred from D-H convention theory [24]. From these parameters, equation (1) [24], and Figure 10, the homogeneous transformation matrices of the proposed system’s each joint could be obtained. According to equation (1), each joint is designated to unique homogeneous transformation matrix.

**Table 1:** **Forward kinematics of the system (D-H parameters)**

Joint	α _***i-***1_	***a***_***i-***1_	***d***_***i***_	θ _***i***_
1	0	0	335	θ_1_
2		75	0	
3	0	270	0	θ_*3*_
4		90	295	θ_*4*_
5		0	0	θ_*5*_
6		0	296	
7		0	0	θ_*7*_ *+*
8		58	0	θ_*8*_

Forward kinematics and Denavit-Hartenberg (D-H) parameters of the overall system are defined by Figure 10 and Table 1. The external arm and surgical instrument are executed using several control algorithms.

1

The transformation matrix of the external arm is calculated as a series of multiplication of the *J1-J6*’s homogeneous transformation matrices. The transformation matrix of the surgical instrument is calculated in a similar way (using *J7-J8*’s homogeneous transformation matrices). The above two transformation matrices describe; i) the position & orientation of the external arm’s translational & fulcrum point movements and ii) the surgical instrument’s position & orientation, respectively. The transformation matrix of the overall system is calculated from the multiplication of the above two transformation matrices [24].

## Preliminary test

### Gripping force

#### Gripping force system setup

**Figure 11:**
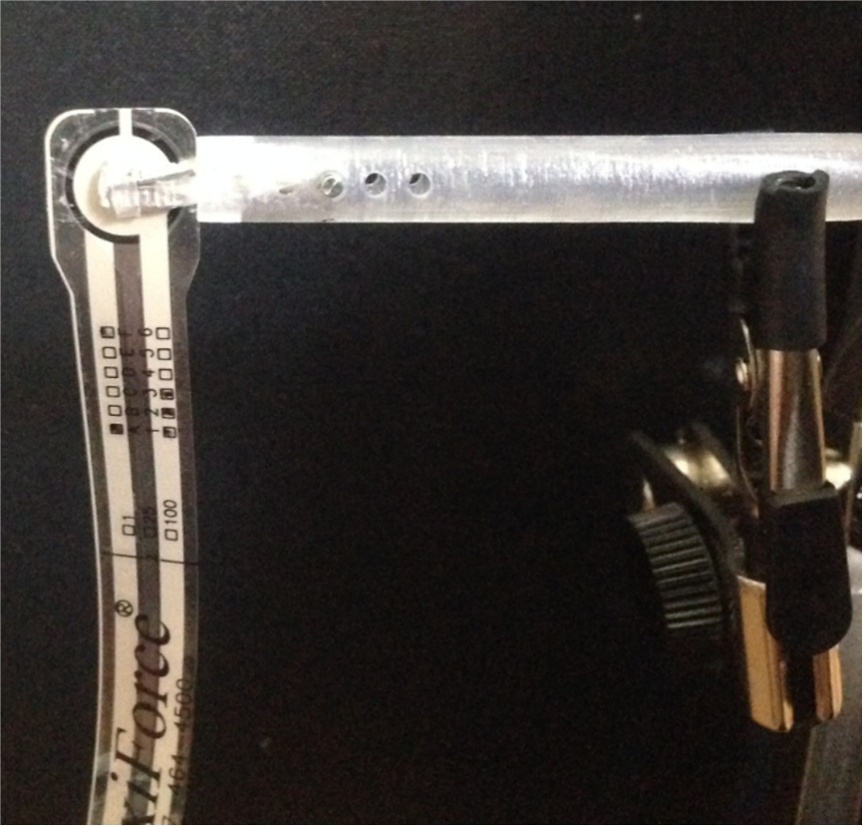
**Gripping force measurement experimental setup using Flexiforce.**

The gripping force was measured using a flexible piezoresistive sensor (Flexiforce, Tekscan Inc., South Boston, MA, USA), as shown in Figure 11. Flexiforce is widely used for pressure measurement in medical applications, and its linearity has been demonstrated [25]. The gripper can be closed by the force generated by Newton’s 3rd law as the inflated catheter balloon pushes the outer shell. To estimate the relationship equation (2) between Flexiforce’s output value and force value, six precision weights (50 g, 100 g, 200 g, 500 g, 1 kg, and 2 kg) were placed on the Flexiforce in order and the output voltages values were measured and converted into force values through a specific LabVIEW® algorithms.

2

The output voltages of Flexiforce were recorded using a data acquisition board (USB-6212 DAQ, National Instruments, Austin, TX, USA). The initial data of 500 samples were used for sensor calibration and initialization in each experiment. For filtering spiky noise, Savitzky-Golay filtering was applied to the signal processing [26, 27]. Signal processing was performed after the gripping force measurement experiment using MATLAB (MathWorks, Natick, MA, USA, using Seoul National University Academic License).

The gripper which was manufactured from the existing stainless forceps (AE-4520-1, KASCO, Sialkot, Pakistan) with the modification on the size and the hole for connecting the gripper to the surgical instrument. In general, medical forceps has the restoring force which has tendency to keep the gripper opened. With our compressor being used in this study, it varied in elastic deformation and was extremely difficult for making the plastic deformation status for forceps. Actually, gripper’s restoring force became smaller as the tips of gripper became larger (in this case, displacement became larger). As a result, the gripping force ('force by catheter balloons’ minus 'restoring force of gripper’) became larger because force by catheter balloon was constant, which meant that the force suggested in this study (displacement between tip is 0) was the smallest force that could be made in this system. In the experiment, assumed that the thickness of Flexiforce and tissue were both thin, the force would be also similar.

#### Relationship between compressor’s pressure and gripping force

**Figure 12:**
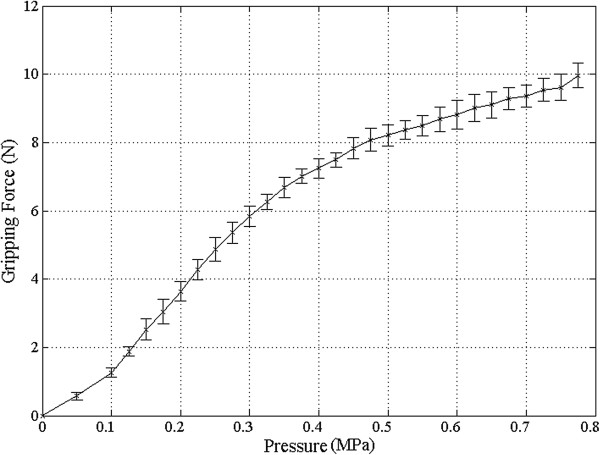
**Experimental result of gripping force in accordance with pressure during 10 repetitions.** The standard deviation of the gripping force was 0.1 N between 0 and 0.775 MPa with 0.025 MPa intervals.

The gripping force was measured 10 times for 0 to 0.775 MPa (interval: 0.025 MPa), and the results are plotted in Figure 12. The standard deviation of the 10 measurements for each pressure was calculated and plotted in Figure 12 as the error bar. The mean of all gripping forces’ standard deviation was computed as 0.1 N. In Figure 12, the pressure section can be divided into two sections except for 0.05 MPa—section 1 (0.1 ~ 0.35 MPa) and section 2 (0.375 ~ 0.775 MPa)—by linearity. Equations (3) and (4) were derived. In sections 1 and 2, the gripping forces from the surgical instrument’s gripper (*GF1* and *GF2*) were determined by pressure values from the compressor according to (3) and (4), respectively.

34

The coefficients: *c*_1_, *i*_1_, *c*_2_ and *i*_2_ of equation (3) and (4) were calculated as 2.2000, -0.7979, 0.6785, and 4.6910, respectively. The means of the differences between the linear equations ((3) and (4)) and the experimental results in Figure 12 were 0.0938 N (standard deviation: 0.0665 N) and 0.0927 N (standard deviation: 0.0607 N) for sections 1 and 2, respectively. These mean values were within the total mean’s standard deviation. This means that the above two equations can be inferred as significant results. These values were referred to in the other experiments conducted in this study.

#### Reaction time

**Figure 13:**
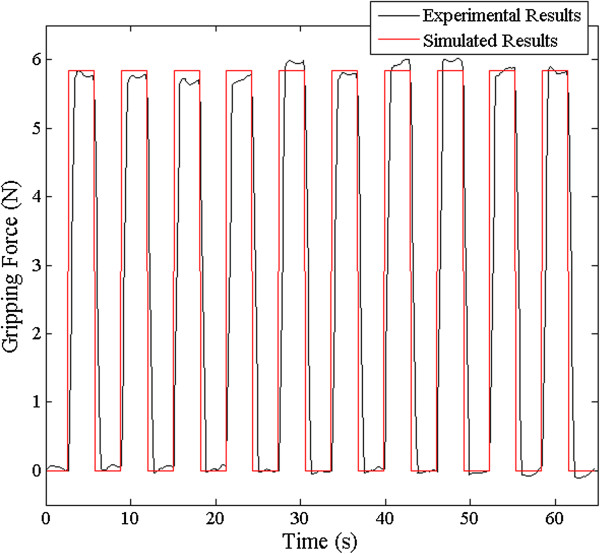
**Experimental results versus simulated results.** The step function of the simulated result was similar to Figure 12’s experimental result for the gripping force at a pressure of 0.3 MPa.

The simulated results were determined by a step function using (3) at 0.3 MPa to be 5.8 N, as shown in Figure 13. Because setting the pressure value as an experimental variable was meaningless for the purpose of the reaction time experiment, a 0.3 MPa was chosen as a representative value. The ideal step function was co-plotted with the experimental results filtered by the Savitzky-Golay filter. This experiment was automatically conducted using a specific LabVIEW® algorithm for excluding users’ irregular HOTAS triggers and repeating the same trigger time. The experimental and simulated results showed close agreement. Compared with the rise time of the gripping force and the time for which the trigger is actually On, the time delay was calculated as 0.4 s.

#### Durability test

For checking the durability of gripping system, the automatic trigger repeating algorithm was performed by repeating On/Off every 1 s for 1,000 gripping motions. This experiment was also conducted at the representative value of 0.3 MPa for the same reason of the reaction time experiment. Table 2 presents the results of this experiment. The repeating experimental value was 5.8 N (SD: 0.2 N) compared with the reference value of 5.8 N (SD: 0.3 N). This result was within the standard deviation. In addition, the standard deviation of the repeating value decreased significantly compared to the reference value because of 1,000 repetitions.

**Table 2:** **Repeated gripping experiment at pressure of 0.3 MPa**

	Mean (N)	Standard deviation (N)
Reference value (from Equ. (3))	5.8	0.3
1,000 times repeated value	5.8	0.2

### Simple peg task

**Figure 14:**
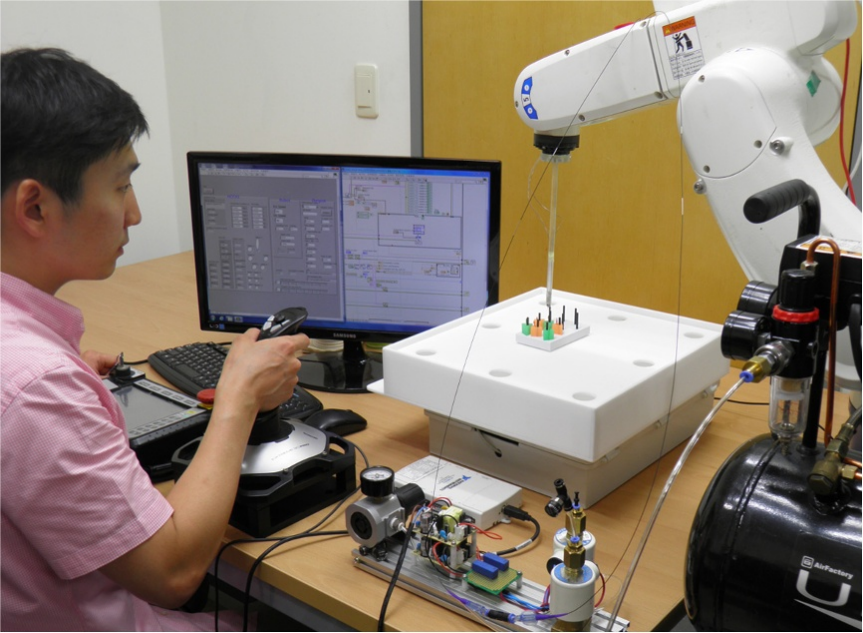
**Block transfer task.** Peg task performed using fundamental of laparoscopic surgery (FLS) task.

To evaluate the proposed surgical robot system, a block transfer task was performed as shown in Figure 14. This task was achieved using the fundamentals of laparoscopic surgery (FLS) peg transfer kit. The simple peg tasks were intended to measure the surgeon’s technical skills and eye-hand coordination during basic laparoscopic surgery and to validate the surgical robot system’s performance [8, 28]. These research followed the FLS curriculum alike our experiment and the time limit was set at 300 s [8, 28, 29]. FLS curriculum is: (a) five novice volunteers were recruited for the experiment using the surgical instrument (b) these volunteers were asked to lift six objects on the left side of the board and to transfer these object to the right side of the board (c) the time for the peg task began when the volunteer grasped the first peg and ended upon the release of the last peg. These volunteers repeatedly performed three trials. According to Table 3, the mean time for the peg task was 176 s. No one exceeded the cut-off time of 300 s in all trials. These results were found to be slightly long in comparison with the results using da Vinci research kit (dVRK), donated by Intuitive Surgical Inc. [30]. For same experimental environment, only one master tool manipulator (MTM) and one patient side manipulator (PSM) of dVRK were used. In the same curriculum for FLS, same volunteers were recruited to carry out the same task. Although amount of reduction time differed from volunteer to volunteer, the peg task’s execution time of 48 ~ 81 s was decreased when it compared with the proposed system’s results. The standard deviation was smaller and more uniform than the proposed system’s results. The volunteer 3 dropped the peg during the task which resulted in creating larger workspace and extra-long execution time. Except for this case, other volunteers showed better performance as they adapted to the system.

**Table 3:** **Execution time of block transfer task**

	Trial number	Volunteer 1	Volunteer 2	Volunteer 3	Volunteer 4	Volunteer 5	Total mean
SOBW	1	255	222	172	154	241	209
	2	221	192	115	153	174	171
	3	148	169	125	122	181	149
	Mean	208	194	137	143	199	176
	SD	45	22	25	15	30	27
dVRK	1	148	132	73	90	147	118
	2	167	108	66	98	131	114
	3	97	100	129	84	126	107
	Mean	137	113	89	91	135	113
	SD	30	14	28	6	9	4

Abbreviation: standard deviation (SD), surgical-operation-by-wire (SOBW), da Vinci research kit (dVRK).

### Workspace

**Figure 15:**
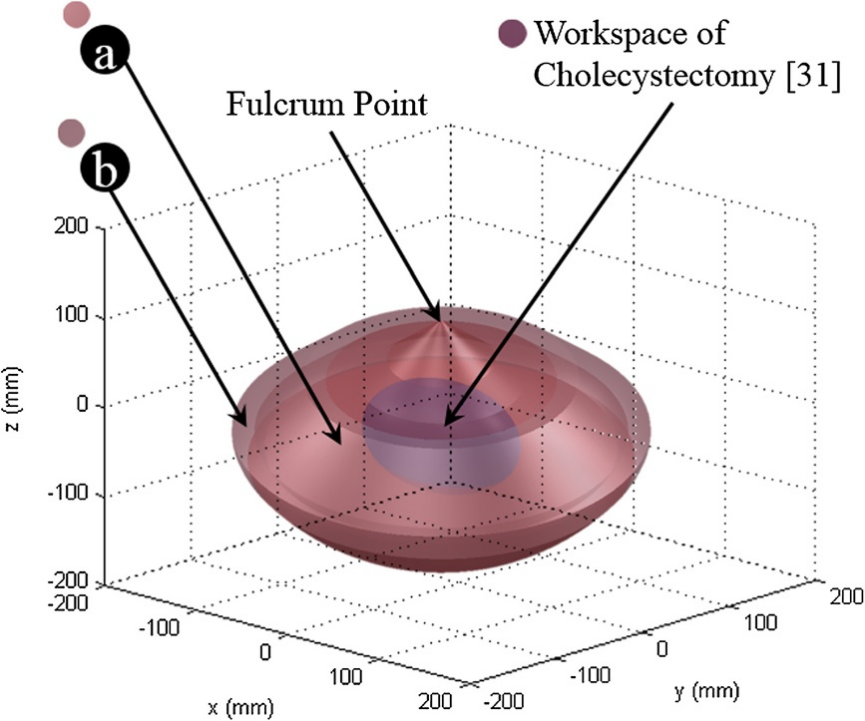
**Workspace of the proposed surgical robot system. (a)** Elbow joint (J7) was considered with external arm (J1-J6). **(b)** Elbow and wrist joints (J7 and J8) were considered with external arm (J1-J6).

Figure 15 shows the calculated workspace. The workspace requirements for a robotic-assisted cholecystectomy were used to validate the proposed surgical robot system [31]. The driving range of each joint was considered using D-H parameters of the proposed system and surgical instrument’s information as shown in Table 1 and Figure 4. The workspace of entire joints satisfied more than 100% of the requirements for cholecystectomy. The workspace was calculated as 11,157.0 cm^3^ which surpassed the 549.5 cm^3^ of the reference’s result [31].

## Discussion

The gripping force predictive linear equations (3) and (4) could provide the gripping force for the pressure range of 1 to 0.775 MPa with 0.025 MPa intervals. The slope of equation (3), *c*_1_ was greater than the slope of equation (4), *c*_2_. Two sections were used owing to air saturation of the catheter balloon. Limited to the diameter of the catheter balloon, it was difficult to generate a greater force for a pressure of 0.35 MPa. The proposed gripping system is remarkable in terms of its decoupling with other joint movements. The value of the gripping force was in close agreement with those of numerous studies [10, 32, 33]. It is expected that greater gripping force will be generated at higher pressures.

According to Figure 13, the experimental and ideal simulated results showed good agreement. The time delay of 0.4 s occurred in passing the pneumatic system, consisting of the SVs, pressure/speed controller, and pneumatic tubes. This means that the pneumatic gripping system reacts to the surgeon’s intention in 0.4 s, enabling almost real-time control.

A repeated gripping experiment indicated the durability of the surgical robot’s instrument. Despite 1,000 repetitions, the gripping force was not affected. This result addressed that surgical instrument’s gripper was greatly durable for many open/close cycles. Although the proposed gripper was not directly compared with da Vinci’s EndoWrist which needed to be discarded after 5 ~ 10 surgeries, it could present a new approach to the next-generation surgical robot’s end-effector for cost effective and reliable surgery. However, like a da Vinci EndoWrist, the successful development of the proposed surgical robot’s gripper should consider sterility issue. Thus, modifying the proposed gripper with the outer shell made of stainless steel and studying the sealing issue are planned in the future.

The simple peg task results were fairly short in comparison to those of other similar studies using same FLS curriculum and FLS kit [8, 28]. It is inferred that the proposed surgical robot system shows good performance and effectiveness for laparoscopic surgery. Most of the results were shorter than those of previous trials. This means that the novice volunteers quickly adapted to the surgical robot system and showed different performances depending on their ability. However, mean of peg task’s execution time and standard deviation were slightly longer compared with the results using dVRK. The major cause of these results was the slow moving velocity of external arm and surgical instrument. This could be overcome by the improvement of the proposed system’s stable control in high speed.

The trajectory of the proposed surgical robot system made a cone shape around a fulcrum point as shown in Figure 15. The status of straight surgical instrument’s reachable workspace (not bended by elbow and wrist joints) was extended by translational movement of external arm. The region of the Figure 15-(a) and (b) was calculated by considering elbow joint movement and elbow & wrist movement, respectively. The proposed surgical robot system would be applicable to other many surgeries covering the cholecystectomy because of its larger workspace. It is even possible to obtain much larger workspace than the current workspace when expanding the movable range of predefined external arm’s limits.

The iHOTAS controller with a 6-axis force/torque sensor sensed the surgeon’s intention of translational movement. It could help in developing a force feedback system. The 6-axis force/torque sensor information, being recorded in real-time, could be analyzed to determine the intent of the surgeon.

Based on the improved feature of the proposed system, SOBW concept, iHOTAS control interface, and novel pneumatic gripping system could be a substitution for other previous surgical robot system developed using mechanical strings and other mechanical parts.

## Conclusions

Recently robotic laparoscopic surgery has been widely used due to its many benefits. However, in existing laparoscopic surgical robots, the end-effector’s gripping joint is coupled with other joints. It could reduce or increase the gripping force according to its posture. This is mainly caused due to the mechanical strings of the existing surgical robot system. In this study, a surgical instrument with a pneumatic gripping system and pitching/yawing joints using micro motors was developed for SOBW. As FBW system was commercially succeed in aerospace engineering, removing the all mechanical strings and directly actuating joint will solve many surgical robot system’s problems: non-uniform gripping force, less durability, and other limitations. This instrument was used to perform a simple peg task with a 6-axis external arm by surgeon’s control using an iHOTAS controller. A gripping force measurement experiment and block transfer task were conducted. To evaluate the proposed system’s clinical applicability, the workspace was calculated. Based on these results, the proposed system is expected to be widely used for laparoscopic robotic surgery. Despite the proposed surgical robot system’s applicability, some improvements are needed. It contains some fragile parts because it was manufactured using a 3D printer’s synthetic resins. To ensure reliability, the prototype surgical instrument should be manufactured using solid materials. In addition, the proposed surgical instrument should resolve sterility issue. Thus, modifying the surgical instrument with the outer shell made of stainless steel and studying the sealing issue will be needed in the future. Then, clinical issues are planned to be considered as a future study, too. Furthermore, a force feedback system should be added using an iHOTAS controller with force sensors.

## Consent

Written informed consent was obtained from the participant for publication of this report and any accompanying images.

## Abbreviations

MIS: Minimally invasive surgery
SOBW: Surgical-operation-by-wire
DOF: Degrees of freedom
FLS: Fundamentals of laparoscopic surgery
HOTAS: Hands-on-throttle-and-stick
iHOTAS: improved HOTAS
FBW: Fly-by-wire
AC: Alternative current
SV: Solenoid valves
D-H: Denavit-Hartenberg
DAQ: Data acquisition board
SG: Spur gear
SBG: Spur and bevel gear
BG: Bevel gear
SD: Standard deviation
dVRK: da Vinci research kit
MTM: Master tool manipulator
PSM: Patient side manipulator.
